# Personalia

**Published:** 1914-05-09

**Authors:** 


### Personalia.

Mr. Reginald George Riches has been appointed to be sixth assistant medical officer at Horton Mental Hospital.

Mr. Cyril Cobb, Mr. H. V. Rowe, Mrs. Herbert Curteis, Lady St. Helier, Mr. W. Lloyd-Taylor, Sir John McDougall, and Mr. P. E. Pilditch have been appointed to serve on the London County Council's sub-committee for the management of Hanwell Mental Hospital.

The following lady and gentlemen will serve on the London County Council's sub-committee for the control of Bexley Mental Hospital during the coming year : Mr. Cyril Cobb, Mr. H. V. Rowe, Mr. T. Hunter, Mr. E. A. H. Jay, Mr. W. Lloyd-Taylor, Mr. W. A. Nicholls, Lady St. Helier, Mr. A T. Taylor, and Lord Windsor.

